# Toxoplasmosis in a patient who was immunocompetent: a case report

**DOI:** 10.1186/1752-1947-5-16

**Published:** 2011-01-18

**Authors:** Aneta K Taila, Ameet S Hingwe, Laura E Johnson

**Affiliations:** 1 Department of Pharmacy, Henry Ford Hospital, Detroit, Michigan, USA; 2 Division of Infectious Diseases, Henry Ford Hospital, Detroit, Michigan, USA

## Abstract

**Introduction:**

*Toxoplasma gondii *is an obligate intracellular protozoan that infects up to one-third of the world's population. Although this case is not the first of its kind, it is clinically important since it will help doctors keep a broad differential diagnosis in mind when attending to similar patients.

**Case presentation:**

We present the case of a 20-year-old man of Middle Eastern heritage presenting with only generalized lymphadenopathy who was diagnosed with acute toxoplasmosis.

**Conclusion:**

This case illustrates the important fact that toxoplasmosis can present with just simple lymphadenopathy, and thus can be confused with other infections such as Epstein-Barr virus and other mononucleosis-like illnesses such as cytomegalovirus, HIV with acute retroviral syndrome, cat scratch disease, leishmaniasis and syphilis. This case underlines why appropriate testing should be performed in confusing cases, and helps increase the knowledge about the diagnosis of this disease.

## Introduction

*Toxoplasma gondii *is an obligate intracellular protozoan that infects up to one-third of the world's population. Human beings can be infected with *T. gondii *by ingestion of tissue cysts in the undercooked meat of intermediate hosts, especially pork and lamb, or by the ingestion of water or food contaminated by feces containing oocysts from the definitive host, members of the feline family [1]. Toxoplasmosis can present with varied signs and symptoms, of which asymptomatic lymphadenopathy is the most common. We present the case of a patient presenting with generalized lymphadenopathy diagnosed as having acute toxoplasmosis. As there are already many examples in the literature detailing the history of toxoplasmosis, this case report is intended to reinforce the clinician's knowledge of the disease and its presentation, especially given its prevalence and the potential consequences of infection.

### Case presentation

A 20-year-old previously healthy man, a student by occupation and a non-smoker not on any medications, presented to his primary care physician with a history of swollen glands for a 'couple of months'. On further review it was found that for one month prior to presentation, our patient had noticed multiple enlarged cervical, occipital, and right inguinal lymph nodes. No constitutional symptoms were reported. Our patient was of Middle Eastern heritage, but was born and raised in the USA. He had not travelled recently, nor had he had any recent contact with sick people or any occupational exposure. On physical examination, our patient was afebrile with normal vital signs. Enlarged, non-tender, freely mobile bilateral cervical and occipital lymph nodes were palpable and measured up to 4cm. His right inguinal lymph nodes were similarly enlarged. The left palatine tonsil was slightly erythematous and enlarged. A monospot test was negative for Epstein-Barr virus infection. Given these findings, the primary care physician prescribed a course of antibiotics for a possible infectious etiology consisting of a three-day course of azithromycin followed by amoxicillin-clavulanate one week later due to persistent symptoms. Initial investigative tests showed normal blood counts and serum electrolytes. An HIV antibody enzyme-linked immunosorbent assay (ELISA) test was also negative.

Our patient returned to the clinic for re-evaluation. With the exception of the enlarged lymph nodes, he remained otherwise clinically asymptomatic. On physical examination, the lymph nodes appeared unchanged, and there were no newly involved nodal chains. Upon more thorough investigation, our patient indicated that approximately once month ago he ate raw kibbe, a Middle Eastern dish that consists of spiced uncooked beef or lamb with grains. Additional laboratory studies were ordered and are listed in Table 1. Our patient was diagnosed with acute toxoplasmosis and counseled regarding dietary habits and risk factors. No specific treatment was administered, and close follow-up was planned to ensure resolution of the lymphadenopathy.

**Table 1:** Follow-up laboratory data

Test	Results
Epstein-Barr virus capsid and nuclear antibody	Negative

Toxoplasma IgG antibody	558IU/mL (positive)

Toxoplasma IgM antibody	Positive

Cytomegalovirus IgM antibody	Negative

Rapid plasma reagin	Negative

## Discussion

Infection of humans with *T. gondii *is common worldwide, with the prevalence varying according to environment, eating habits, and age [2]. Contact with this obligate intracellular protozoan occurs through direct ingestion of food or water contaminated with cat feces containing oocysts, ingestion of tissue cysts in uncooked meat, transplacental infection of the fetus, white blood cell transfusion or organ transplantation. Our patient was probably exposed to *T. gondii *by eating raw lamb. Prior case reports have shown that the disease has a higher prevalence among men (79% versus 63.4% in women) and that age-dependent seroprevalence reaches > 92% in the age 40 to 50 group [3]. In seroepidemiological surveys in the USA, 11% of persons aged 6 to 49 are seropositive for *T. gondii *[4].

Clinical presentation of *T. gondii *infection depends on the age and immune status of the patient. In the majority of patients who are immunocompetent, both adult and pediatric, primary infection is usually asymptomatic. In approximately 10% of this patient group, a non-specific and self-limiting illness is manifested most typically by isolated cervical or occipital lymphadenopathy lasting for less than four to six weeks. The lymph nodes are usually discreet, non-tender, and do not suppurate. Differential diagnoses include Epstein-Barr virus and other mononucleosis-like illnesses including cytomegalovirus and HIV with acute retroviral syndrome. Though not as common, hematological malignancies, cat scratch disease, leishmaniasis and syphilis can also cause lymphadenopathy. Very infrequently immunocompetent hosts might also suffer from myocarditis, polymyositis, pneumonitis, hepatitis, or encephalitis. After the acute phase, almost all patients will remain chronically infected with tissue cysts that are dormant and cause no clinical symptoms.

In contrast, toxoplasmosis in patients who are immunocompromised can be a life-threatening infection. In this population, toxoplasmosis almost always occurs as a result of reactivation of chronic disease and most typically affects the central nervous system. Toxoplasmic encephalitis has a varied clinical presentation, ranging from an acute confusional state with or without focal neurological deficits evolving over days to a subacute gradual process evolving over weeks. Other presentations of toxoplasmosis in patients who are immunocompromised include chorioretinitis, pneumonitis, or multi-organ failure.

Diagnosis of *T. gondii *infection can be made via a number of methods, both directly via polymerase chain reaction (PCR), hybridization, isolation, and histology and indirectly via serological methods. In our patient, serology was helpful. In patients who are immunocompetent, indirect serological methods are more widely used as they are readily available, faster, and cheaper. However, testing for IgG antibodies to *T. gondii *should also be performed in asymptomatic patients who are immunocompromised, as this allows identification of those at risk for reactivation of latent infection. Additionally, an absence of IgG antibodies in pregnant women allows identification of those at risk of acquiring infection during gestation.

Serological methods used to detect antibodies include the Sabin-Feldman dye test, immunofluorescent antibody test, ELISA, IgG avidity test, and agglutination tests. Assays for functional affinity of these antibodies have become standard as they help discriminate between recently acquired and more chronologically distant infections. The presence of high avidity antibodies essentially excludes infection acquired in the past three to four months; however, low avidity antibodies may persist beyond three months of infection and therefore do not necessarily indicate recent infection [5].

In patients who are immunocompromised, direct methods of detection must be employed. Body fluids and tissues can be subjected to PCR amplification of *T. gondii *genes (specifically, the B1 gene). Assuming appropriate sample collection, handling, and storage, sensitivity is no greater than 50% but highly specific [6]. Isolation of *T. gondii *directly from blood or body fluids is indicative of acute infection, whether newly acquired or reactivation of latent infection. Direct diagnosis can also be made with tissue sections or body fluid smears that demonstrate tachyzoites.

Treatment with pyrimethamine, sulfadiazine and folinic acid is usually reserved for patients who are immunocompromised and those patients who are immunocompetent who have severe or persistent symptoms. Duration of treatment varies from two to four months depending upon resolution of clinical signs and symptoms. Alternatively, trimethoprim/sulfamethoxazole is equivalent to pyrimethamine/sulfadiazine [7]. Maintenance therapy should be started after the acute phase has resolved and should consist of the same regimen as in the acute phase but at half dose. This should continue for the life of the patient or until the immunosuppression has resolved [8].

## Conclusion

We present a case of acute toxoplasmosis manifesting as generalized lymphadenopathy with the leading risk factor in this case being the consumption of raw meat. For the general internist, a broad differential should be kept in mind when patients present with lymphadenopathy and appropriate testing should be performed. When the diagnosis is made, treatment is rarely required for asymptomatic patients who are immunocompetent. Proper education and counseling regarding risk factors can reduce the incidence and risk of acquiring the infection.

## Consent

Written informed consent was obtained from the patient for publication of this case report. A copy of the written consent is available for review by the Editor-in-Chief of this journal.

## Competing interests

The authors declare that they have no competing interests.

## Authors' contributions

AT, AH and LJ had equal role in writing the manuscript. All authors read and approved the final manuscript.
